# The mitochondrial genome of the assassin bug *Sclomina erinacea* (Hemiptera: Reduviidae)

**DOI:** 10.1080/23802359.2019.1667272

**Published:** 2019-09-20

**Authors:** Qian Zhao, Ping Zhao, Hu Li, Wanzhi Cai, Fan Song

**Affiliations:** a Department of Entomology and MOA Key Lab of Pest Monitoring and Green Management College of Pant Protection, China Agricultural University, Beijing, China;; b Guangdong Key Laboratory of Animal Conservation and Resource Utilization, Guangdong Public Laboratory of Wild Animal Conservation and Utilization, Guangdong Institute of Applied Biological Resources, Guangzhou, China

**Keywords:** Mitochondrial genome, Reduviidae, Harpactorinae, *Sclomina erinacea*

## Abstract

The complete mitochondrial genome (mitogenome) of the assassin bug, *Sclomina erinacea* Stål, was determined in the present study. The sequenced mitogenome is a typical circular DNA molecule which is 15,828 bp in length, containing 13 protein-coding genes, two rRNA genes, 22 tRNA genes and a putative control region. All protein-coding genes are initiated by ATN codons and terminated by TAA or TAG codons except *COII, COIII* and *ND4* which use a single T residue as the stop codon. All tRNAs have the cloverleaf structure with the exception of *tRNA^Ser(AGN)^* and the length of them range from 62 to 70 bp. The control region is 818 bp long with an A + T content of 67.1%. The phylogenetic analysis supports the monophyly of Harpactorinae and *Sclomina erinacea* is the closest relative to *Macracanthopsis nodipes*.

The assassin bug Harpactorinae (Hemiptera: Reduviidae) is the largest subfamily of Reduviidae distributed around the world mainly in tropical and subtropical regions. The Harpactorinae currently contains about 180 species in China, occurring in the southern region of China. *Sclomina erinacea,* which belongs to the subfamily Harpactorinae, is a widely distributed predatory natural enemy insect in China and can prey on a variety of agricultural pests (Zhao and Liu 2015). In this study, the complete mitogenome of *Sclomina erinacea* is sequenced and annotated for the first time. The samples were collected in Meiling, Jiangxi, China (28°48′39.0″N 115°41′52.1″E). Voucher specimen was deposited at the Entomological Museum of China Agricultural University (No. VCim-00168) and the sequence has been submitted to GenBank (Accession number: MK696614).

The mitogenome is a single circular DNA molecule of 15,828 bp in size that encode 37 genes (13 protein-coding genes, 22 tRNA genes, and two rRNA genes) and a control region. Gene order is identical to the putative ancestral gene arrangement of insect (Cameron 2014; Song et al. 2016a; Li et al. 2017; Liu et al. 2019). Except control region, this mitogenome has 3 inter-genic regions > 90 bp. A 126 bp noncoding region could be found between *tRNA^Cys^* and *tRNA^Tyr^* and a 94 bp noncoding region exist between *CytB* and *tRNA^Ser (UCN)^*. Another 218 bp noncoding region is located between *tRNA^Ser(UCN)^* and *ND1*, which has also been found in other assassin bugs (Sun et al. 2019). There are totally 51 bp overlapped nucleotides between neighboring genes in 12 locations, ranging from 1 to 14 bp in size.

The nucleotide composition of the whole mitogenome shows highly A + T biased. The A + T content is 71.6% with positive AT-skew (0.14) and negative GC-skew (–0.17). All protein-coding genes initiate with ATN as the start codon. The stop codon TAA terminates 7 protein-coding genes and TAG terminates 3 protein-coding genes. Whereas *COII, COIII* and *ND4* use a single T residue as incomplete stop codon which has repeatedly found in other insect mitogenomes (Song et al. 2016b; Liu et al. 2018; Sun et al. 2019).

The length of the 22 sequenced tRNA genes range from 62 to 70 bp and most tRNA genes could be folded into the typical cloverleaf structure except for *tRNA^Ser (AGN)^*, due to the deficiency of the dihydrouridine (DHU) arm which is typical feature of insect mitogenomes (Jiang et al. 2016). The *lrRNA* is 1,267 bp in length with an A + T content of 74.1% and the *srRNA* is 778 bp in length with an A + T content of 72.4%. The control region, which is located between *srRNA* and *tRNA^Ile^*, is 818 bp long and is biased toward A + T (67.1%).

Phylogenetic tree based on the maximum likelihood method shows Harpactorinae is monophyletic (Fgure 1), which is also recovered in previous molecular and morphological analyses (Weirauch et al. 2014; Liu et al. 2019). The sister relationship between *Sclomina erinacea* and *Macracanthopsis nodipes* is also highly supported (bootstrap = 100).

## Figures and Tables

**Figure 1.**
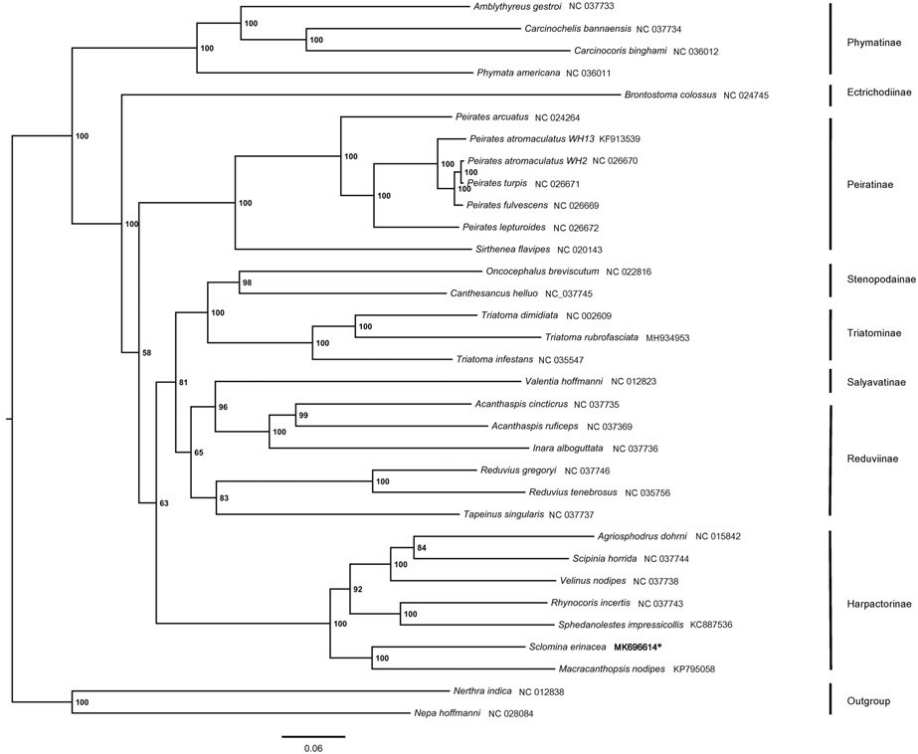
Phylogenetic relationship of *Sclomina erinacea* and other 32 species among Reduviidae. Phylogenetic tree was inferred from ML analysis of the 13 protein-coding genes and two rRNAs genes (12,812 bp) and generated by IQ-TREE 1.6.5 (Trifinopoulos et al. 2016) under the GTR + I + G model. The number beside the nodes are percentages of 1000 bootstrap values. The GenBank accession number of the species is shown behind the species name. The newly sequenced mitogenome is highlighted by the asterisk.
